# Electroacupuncture and Transcutaneous Electrical Nerve Stimulation Induced Sensations in Bell’s Palsy Patients: A Quantitative Current Intensity Analysis

**DOI:** 10.3389/fnins.2021.692088

**Published:** 2021-07-09

**Authors:** Han Cui, Haibo Yu, Xingxian Huang, Lixiong Wu, Weizheng Zhong, Yanhua Gou, Xuemei Cao, Yongfeng Liu, Yuanyuan Hong, Shaoyun Zhang, Minmin Zhan, Guanglin Li, Zhuoxin Yang

**Affiliations:** ^1^Department of Acupuncture and Moxibustion, Shenzhen Traditional Chinese Medicine Hospital, Shenzhen, China; ^2^CAS Key Laboratory of Human-Machine Intelligence-Synergy Systems, Shenzhen Institutes of Advanced Technology, Chinese Academy of Sciences, Shenzhen, China; ^3^The fourth Clinical Medical College, Guangzhou University of Chinese Medicine, Shenzhen, China

**Keywords:** electroacupuncture, Bell’s palsy, Facial Nerve, finite element model analysis, transcutaneous electrical nerve stimulation

## Abstract

**Background:**

The intensity of electrical acupoint stimulation such as electroacupuncture (EA) and transcutaneous electrical nerve stimulation (TENS) is regulated by the observation of skin shivering or the participant’s comfort response. However, the specific intensity and spatial scope following EA or TENS stimulation are unclear.

**Objective:**

This study aimed to test the stimulatory current intensities of lower and upper sensation thresholds in TENS- and EA-based treatment of Bell’s palsy patients. Also, the spatial scope of the stimulation at these current intensities was simulated and measured quantitatively.

**Methods:**

A total of 19 Bell’s palsy patients were recruited. Six acupoints on the affected side of the face were stimulated by TENS and EA successively at 30-min intervals. During the stimulation, the current intensity was regulated gradually from 0 to 20 mA, and we simultaneously measured the lower (sensory) and upper (tolerability) sensations. After the treatment by TENS and EA, the modified Chinese version of the Massachusetts General Hospital Acupuncture Sensation Scales (C-MMASS) was applied to survey the de-qi sensations during stimulation. Additionally, we analyzed the correlation between current intensities and C-MMASS and comfort scores. Finite element models were established to depict the spatial distribution of electric field gradients at the lower and upper thresholds.

**Results:**

The mean sensory and tolerability thresholds of TENS were 3.91–4.37 mA and 12.33–16.35 mA, respectively. The median sensory and tolerability thresholds of EA were 0.2 mA and 2.0–3.2 mA, respectively. We found a significant correlation between total C-MMASS scores and the current intensities at the tolerability threshold of TENS. The finite element model showed that the activated depths of TENS and EA at the lower threshold were 3.8 and 7 mm, respectively, whereas those at the upper threshold were both 13.8 mm. The cross-sectional diameter of the activated area during TENS was 2.5–4 times larger than that during EA.

**Conclusion:**

This pilot study provided a method for exploring the current intensity at which the de-qi sensations can be elicited by TENS or EA. The finite element analysis potentially revealed the spatial scope of the electrical stimulation at a specific current intensity.

## Introduction

According to the theory of Traditional Chinese Medicine (TCM), the clinical efficacy of acupuncture can only be achieved if the manipulation elicits de-qi, a sensation experienced by both the subject and acupuncturist (Kong et al., 2007; Yang et al., 2013; Zhu et al., 2013). However, the feelings of the acupuncturist are more prone to subjective bias. The current investigations on de-qi sensations are focusing more on the subject’s sensations (Yu et al., 2012). The de-qi sensation experienced by the subject encompasses a series of specific sensations such as heaviness, numbness, soreness, fullness, coolness, warmness, etc. (Kong et al., 2007; Yang et al., 2013). The term “de-qi” is vital in the clinical practice of acupuncture. Numerous studies have explored the characterization, quantification, clinical efficacy, and underlying mechanism of de-qi (Leung et al., 2006; Kou et al., 2007; Zhou et al., 2011; Yu et al., 2012; Park et al., 2013; Wang et al., 2013; Yang et al., 2013; Zhang et al., 2013).

In clinics, patients with Bell’s palsy are, in most cases, managed using acupuncture and electroacupuncture (EA) in China (Chen et al., 2010; Teixeira et al., 2011; Uchino, 2013; Xu et al., 2013; Li et al., 2015; Zhang et al., 2019). In a high-quality randomized controlled trial, strengthened acupuncture stimulation could elicit de-qi sensations and demonstrated a better therapeutic effect than sham acupuncture; the inserting needles remained in place (Xu et al., 2013). However, it is difficult to quantify the intensity of manual acupuncture stimulation since the needles are manipulated through lifting, thrusting, and twirling to achieve the de-qi sensation, which potentially differs among acupuncturists. In contrast, acupuncture combined with electrical stimulation (EA) can achieve continuous, stable, and quantitative stimulation without manual manipulation (Yeh et al., 2020; Zhang et al., 2020). In clinical practice, the paired needles connected to the electrical stimulating system in pairs do not need extra manual manipulation in maintaining the sensation after the de-qi sensation has been elicited. In recent years, several high-quality clinical studies had demonstrated the efficacy of EA in functional constipation (Liu et al., 2016), stress urinary incontinence (Liu et al., 2017), and chronic stable angina (Zhao et al., 2019). Also, EA-treated Bell’s palsy patients were characterized by a better prognosis than manual acupuncture-treated patients (Wang et al., 2020). However, the intensity of EA in these clinical studies remained semiquantitative because the current intensity was regulated based on skin shivering or the participant’s comfort response (Liu et al., 2016, 2017; Fei et al., 2019; Zhao et al., 2019). The current intensity was associated with the physiological and therapeutic effects of EA (Ju et al., 2015; Lv et al., 2019; Yu et al., 2019). Therefore, quantification of the current intensity in clinics will guide investigators to elucidate the physiological mechanisms underlying the clinical effects of EA. Such findings will further demonstrate why a particular current intensity is effective in some case but not others, thereby advancing the efficiency of EA stimulation in the future.

In this study, we aimed to evaluate the range of current intensity at which de-qi sensations can be elicited during EA management of patients with Bell’s palsy. The lower and upper limits of sensations elicited by EA were established based on the recordings of sensory and tolerability thresholds. The paralyzed side of the face was stimulated, and the EA was applied on three pairs of acupoints, including Yangbai (GB14)–Taiyang (EX-HN5), Yingxiang (LI20)–Dicang (ST4), and Jiache (ST6)–Quanliao (SI18), which were selected by an experienced acupuncturist aided by literature reports (Xu et al., 2013; Wang et al., 2020). The current intensities at which de-qi sensations can be elicited were included between the range of sensory threshold and tolerability threshold. The sensation thresholds of transcutaneous electrical nerve stimulation (TENS) were also evaluated in this study. McDonnall et al. revealed that TENS sensory thresholds were 0.4 ± 0.1 V, and about 1.6 V exhibited unbearable stimulation (Mcdonnall et al., 2009). Frigerio et al. (2015) reported that the average current intensities were 5.19–12.2 mA from very low-level pain to intolerable pain. Elsewhere, Ilves et al. (2019) demonstrated that 1 mA induced sensible stimulation, and the tolerable stimulation was nearly 7–8 mA. In this study, both EA and TENS were placed on the same acupoints. The characteristics of these thresholds were statistically analyzed of note because the thresholds and the subjective sensations vary from person to person. We were curious to establish whether the current intensities were related to the intensity of de-qi sensations regardless of the individual differences in their perception. To address this issue, the de-qi sensations of EA and TENS were quantified via the modified Chinese version of Massachusetts General Hospital Acupuncture Sensation Scales (C-MMASS) (Yu et al., 2012). Also, comfort is another important indicator that dictates the current intensity of EA and TENS. Therefore, we surveyed the rating score of comfort.

Additionally, finite element analysis was applied to simulate the spatial distribution of electric field gradients at the current intensities tested in the study (Filipovic et al., 2011; Datta et al., 2013). The depth and cross-sectional diameter of the activated spatial extent were measured quantitatively using the simulating results. The de-qi sensations are associated with the activation of Aδ and C fiber (Wang et al., 1985; Beissner et al., 2010). Therefore, the simulation of electric field gradient can help to determine the target fibers in spatial range.

## Materials and Methods

### Subjects

A total of 19 Bell’s palsy patients (13 males, 6 females, age between 14 and 69) from the outpatient department of the Shenzhen Traditional Chinese Medicine Hospital were enrolled in the study. Thirteen patients were affected on the right side of the face, whereas six patients were affected on the left side. The included subjects had no clinical history of other neuromuscular disorders and diabetes mellitus attacking peripheral nerve function. The Ethical Committee of Shenzhen Traditional Chinese Medicine Hospital approved the study (Approval Number: 2017-05). Each subject signed an informed consent. An experienced doctor (over 10 years of clinical experience) assessed the degree of Bell’s palsy patients using the Facial Nerve Grading Scale 2.0 (FNGS 2.0) (Vrabec et al., 2009). The body mass index (BMI) was also computed. The detailed demographic information of the 19 subjects is shown in Table 1.

**TABLE 1:** The demographic information of all the subjects.

ID	Gender	Age (years)	Paretic side	BMI	FNGS score
1	M	40	R	24.2	13
2	M	41	L	24.5	11
3	M	54	R	23.7	5
4	M	23	L	19.6	20
5	F	51	R	22.2	22
6	M	19	L	28.4	18
7	F	25	R	22.3	16
8	M	40	R	28.7	16
9	F	57	R	22.6	8
10	M	34	R	27.1	15
11	M	36	R	26.0	13
12	F	54	R	18.5	24
13	M	48	L	20.8	13
14	M	29	R	26.0	10
15	M	36	R	27.5	14
16	F	24	R	17.1	14
17	M	69	L	23.2	17
18	F	61	R	27.5	10
19	M	40	L	23.5	19

### Equipment and Electrodes

A commercial current control electrical stimulation system (NT6021, Dundex, Shenzhen Dongdixin Technology Co. Ltd., Shenzhen, China) was used for continuous biphasic rectangular stimulation (equal width and amplitude of the positive and negative phases). The pulse width of a single positive or negative phase was 100 μs with a pulse repetition frequency of 20 Hz. The stimulation parameters were the same with the TENS and EA treatment of Bell’s palsy in the outpatient department. We gradually increased the stimulation current intensity from 0 to 20 mA (0.2-mA intervals).

A commercial hydrogel electrode (diameter, 22 mm) (CM22R, Shanghai Hanjie Medical Technology Co. Ltd., Shanghai, China) was applied as the surface electrode in TENS. A stainless steel acupuncture needle (25-mm long and 0.3-mm diameter) (Suzhou Huanqiu Acupuncture Medical Co. Ltd, Suzhou, China) was used for EA. Both the surface electrode and the acupuncture needle were connected to the same electrical stimulation system.

### Experimental Procedure

A digital phone camera was used to record facial behavior, including raising eyebrows, closing eyes, grinning, pouting, and wrinkling nose (maximum of five trials each), for offline FNGS 2.0 assessment. Then subjects were laid facing up for three experimental steps. In the first step, the surface electrode was used for stimulation with a current from 0 mA to the maximum tolerable current (0.2-mA intervals). The following standards were applied:

• Sensory threshold: minimal sensation at the stimulation position.
• Tolerability threshold: unbearable sensation or reaching the maximum stimulation intensity (20 mA).

**FIGURE 1:**
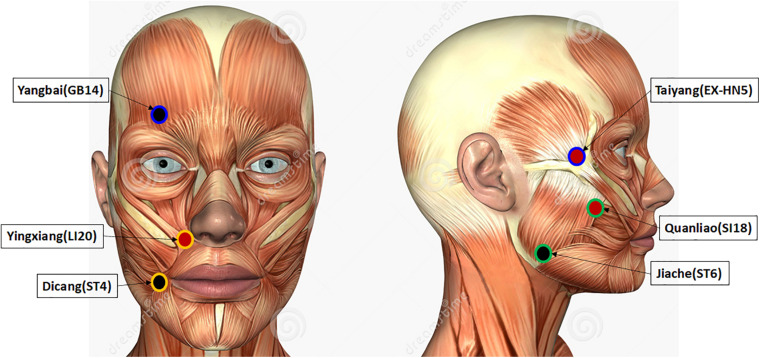
Illustration of the stimulated acupoints. The acupoints colored in red were connected with positive electrodes, and the acupoints colored in black were connected with negative electrodes. Each pair of electrodes was circled with a same color.

The skin of the paralyzed side of the face was cleaned using alcohol cotton stickers before applying the surface electrodes. Six acupoints of the paralyzed side were attached with the surface electrodes, including Yangbai (GB14), Taiyang (EX-HN5), Yingxiang (LI20), Dicang (ST4), Jiache (ST6), and Quanliao (SI18). The acupoint Yangbai was paired with Taiyang (Pair 1), Yingxiang with Dicang (Pair 2), and Jiache with Quanliao (Pair 3) during the stimulation. Taiyang, Yingxiang, and Quanliao were connected with positive electrodes, and Yangbai, Dicang, and Jiache were connected with negative electrodes. The illustration of these acupoints is shown in Figure 1. The red acupoints were connected with positive electrodes, and the black acupoints were connected with negative electrodes. Each pair of electrodes was circled with the same color. The sensory and tolerability thresholds of each electrode pair were separately assessed. The subjects were then treated with TENS at the current intensity of tolerability threshold for 10 min. The C-MMASS was used to evaluate the sensations (Yu et al., 2012). The C-MMASS included 12 descriptors: soreness, aching, deep pressure, heaviness, fullness/distension, tingling, numbness, dull pain, warmth, cold, throbbing, and patient perceptions (Yu et al., 2012). The descriptors were scaled from none (point 0) to unbearable (point 10). Besides, a visual analog scale was applied to assess the overall comfort (1, 9, and 5 denoted very uncomfortable, very comfortable, and neither uncomfortable nor comfortable, respectively).

In the second step, the surface electrodes were removed from the skin surface, and the subject rested for 30 min without any treatment. In the third step, the subject was stimulated with EA. The acupuncture needles were inserted (5-mm deep) at the same acupoints of surface electrodes. The stainless acupuncture needles were connected to the electrical stimulation system via electric wires. The pairs of acupoints in EA stimulation were the same with the TENS stimulation. We then assessed the sensory and tolerability thresholds of each pair separately. After that, the subject was treated with EA at the tolerability threshold for 10 min. C-MMASS and comfort rating scales were applied to evaluate the sensations and comfort.

### Data Analysis

The sensory and tolerability thresholds among different electrode pairs were compared. Shapiro–Wilk test was used for normality analysis of the thresholds. Depending on the distribution whether normal or not, the analysis of variance (ANOVA) and the Kruskal–Wallis H-tests were used, respectively, to compare the significant differences of thresholds among different pairs. Bonferroni method was used to correct the comparisons of each two pairs. The paired *t*-test (normal distribution) or Wilcoxon signed-sum test (skewed distribution) were used to analyze the difference between sensory and tolerability thresholds of one pair. A value of *p* < 0.05 was considered statistically significant.

In the analysis of C-MMASS scores, the sum scores of all subjects in each descriptor were plotted in the form of a stack bar. The length of one color in each bar represented the score of one subject’s sensation. In this study, the distribution of C-MMASS scores and comfort scores were skewed. Therefore, the correlation between these scores and current intensities at tolerability threshold was analyzed by Spearman correlation.

### Transcutaneous Electrical Nerve Stimulation and Eletroacupuncture Modeling

**FIGURE 2:**
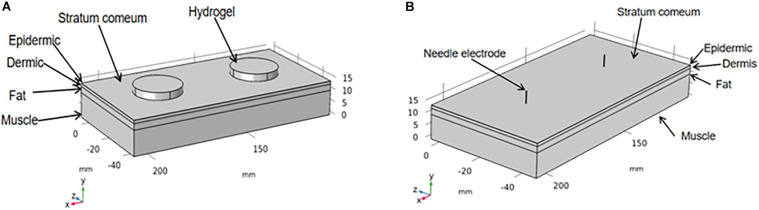
Finite element model of hydrogel electrode **(A)** and stainless steel needle **(B)** on the facial tissues.

Here, a three-dimensional finite element model with different electrodes and facial tissues was established via the COMSOL software (COMSOL Multiphysics 4.3a, COMSOL, Stockholm, Sweden) to evaluate the activation spatial scope of TENS and EA at a certain current intensity. The facial tissue model (2 cm × 7 cm) had five horizontal layers: the stratum corneum, epidermis, dermis, fat, and muscle, as illustrated in Figure 2. The hydrogel electrode used in TENS measured 2.2 cm in diameter and 3 mm in thickness. The stainless steel needle used in EA measured 0.3 mm in diameter and a penetration depth of 5 mm. The thickness of each layer and the electrical parameters of the related materials are shown in Table 2. The thicknesses of the facial layers were defined based on a cadaver study (Ha et al., 2005), whereas the electrical conductivity parameters were set based on the previous measurement of the tissue layers and materials (Gabriel et al., 1996; Sha et al., 2008; Doheny et al., 2010). Both the anode and cathode electrodes were placed on the stratum corneum surface with a separation distance of 5 cm, close to the actual distance in a pair of electrodes. The model was simplified to a stationary electrical field; the ground served as the anode electrode, and the stimulation current source served as the cathode electrode. The electrical stimulation waveform was biphasic rectangular with a single positive or negative phase lasting 100 μs (the ground and source electrodes were alternated every 100 μs).

**TABLE 2:** The electrical conductivity and thickness of each layer in the finite element model.

Layer	Electrical conductivity (S/m)	Thickness (mm)
Stratum	2 ^∗^ 10^–5^	0.029
Epidermis	Horizontal: 0.95, Vertical: 0.15	0.060
Dermis	Horizontal: 2.57, Vertical: 1.62	1.3
Fat	0.04	2.5
Muscle	Horizontal: 0.25, Vertical: 0.75	10
Hydrogel	4.6 ^∗^ 10^–3^	Radius: 11, Thickness: 3
Stainless steel needle	1.74 ^∗^ 10^6^	Radius: 0.15

In the simulation of nerve fiber excitation, an activating mathematical function was often used to approximate extracellular electrical field influence on a specific segment of the nerve fiber (Rattay, 1999). The activating function in the nth compartment of the axon model is expressed as:

(1) $$f_{n}=1/c\,\left(\frac{V_{n-1}-V_{n}}{R_{n-1}/2+R_{n}/2}+\frac{V_{n+1}-V_{n}}{R_{n+1}/2+R_{n}/2}+\ldots \right)$$

where _*f_n*_ is the change in transmembrane potential resulting from an applied stimulus, which is referred to as the activating function, c is the membrane capacity, _*V_n–1*_ , _*V_n*_, and _*V_n+1*_ are the extracellular voltage outside the adjacent compartment n - 1, n, and n + 1 relative to the ground, _*R_n–1*_, _*R_n*_, and _*R_n+1*_ denote the resistance of compartments n - 1, n, and n + 1, respectively. In the simplifications for long fibers of an ideal internode membrane, in which the node-to-node distance and the node length are approaching 0, the activating function is expressed as:

(2) $$f=\frac{d}{4\,\rho \,c}•\frac{\delta^{2}\,V}{\delta \,x^{2}}$$

where d is the fiber diameter, _ρ_ is the axomplasmatic resistivity, c is the membrane capacity, and x is the spatial differential along the fiber. It can be observed from this equation that the activating function is proportional to the second-order spatial difference of the extracellular potential along the fibers. Therefore, the distribution of second-order electrical field gradient simulated by finite element model was used to represent the probability of nerve activation in this study.

## Results

### Sensory and Tolerability Thresholds of Transcutaneous Electrical Nerve Stimulation

**FIGURE 3:**
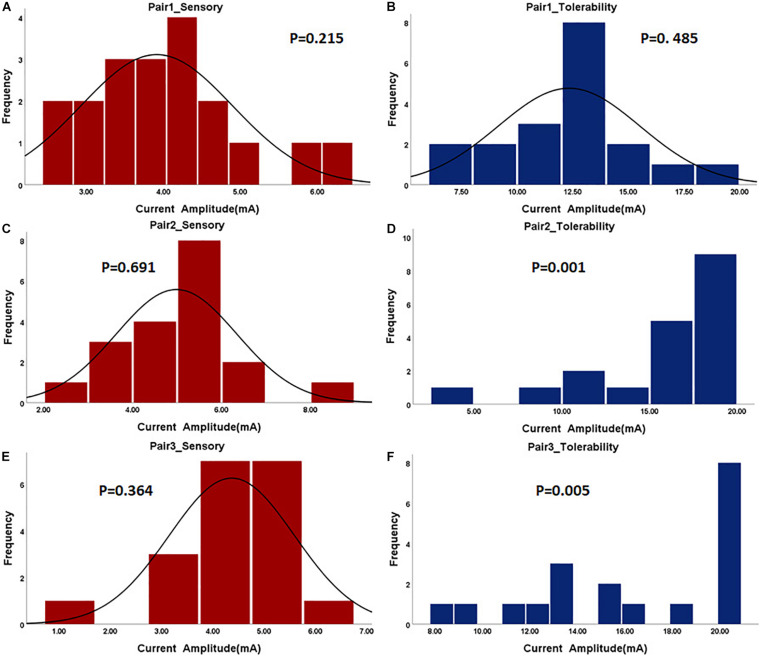
The histogram of sensory and tolerability thresholds of the surface electrode pairs. The *P*-value of the normality test is shown in each figure. P greater than 0.05 was considered as the normal distribution. **(A)** Sensory threshold of Pair 1. **(B)** Tolerability threshold of Pair 1. **(C)** Sensory threshold of Pair 2. **(D)** Tolerability threshold of Pair 2. **(E)** Sensory threshold of Pair 3. **(F)** Tolerability threshold of Pair 3.

**FIGURE 4:**
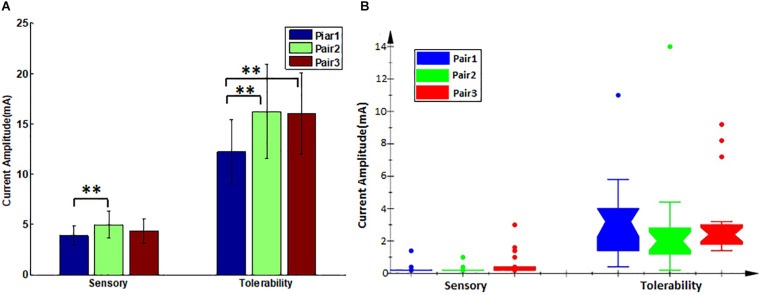
The sensory and tolerability threshold comparisons between the pairs. **(A)** Transcutaneous electrical nerve stimulation (TENS) thresholds, error bars denote the mean and standard deviation of the data, ^∗∗^(*p* < 0.01). **(B)** Electroacupuncture (EA) thresholds, its lower and upper boundary lines are the 25%/75% quantile of the data, outliers are also marked on the plot.

In the first step, the paralyzed side of the face was stimulated by TENS. The distributions of sensory and tolerability thresholds were normal (*p* > 0.05) except for tolerability thresholds of Pair 2 and Pair 3 (Figure 3) because some were attained at the maximum current intensity. Nine of 19 subjects attained tolerability thresholds at the maximum current intensity in Pair 2, and eight of 19 subjects in Pair 3. However, one subject attained tolerability thresholds at the maximum current intensity in Pair 1. Yet, the tolerability thresholds should be greater than the maximum current intensity in such cases. Statistically, the sensory and tolerability thresholds of Pair 1 were 3.91 ± 0.99 and 12.33 ± 3.19 mA, respectively, the sensory and tolerability thresholds of Pair 2 were 4.99 ± 1.36 and 16.35 ± 4.71 mA, respectively, the sensory and tolerability thresholds of Pair 3 were 4.37 ± 1.22 and 16.12 ± 4.09 mA, respectively. The tolerability thresholds were significantly greater than the sensory thresholds in each pair. Besides, the sensory and tolerability thresholds were significantly different between the pairs (Table 3). The mean sensory thresholds of Pair 2 were greater significantly than Pair 1 (*p* = 0.008). Pair 2 and Pair 3 had significantly greater mean tolerability threshold values than Pair 1 (*p* = 0.003 and *p* = 0.006, respectively). The results are shown in Figure 4A.

**TABLE 3:** The statistical sensory and tolerability threshold results of the transcutaneous electrical nerve stimulation (TENS’s) electrode pairs.

Threshold (mA)	Pair 1 (mean ± SD)	Pair 2 (mean ± SD)	Pair 3 (mean ± SD)	*p*-value
Sensory	3.91 ± 0.99	4.99 ± 1.36	4.37 ± 1.22	0.028Δ
Tolerability	12.33 ± 3.19	16.35 ± 4.71	16.12 ± 4.09	0.005◆
*p*-value	<0.001	<0.001	<0.001	

**Δp = 0.008, Pair 1 vs. Pair 2; Bonferroni corrected. ◆p = 0.003, Pair 1 vs. Pair 2; p = 0.006, Pair 1 vs. Pair 3; Bonferroni corrected.**

### Sensory and Tolerability Thresholds of Electroacupuncture

**FIGURE 5:**
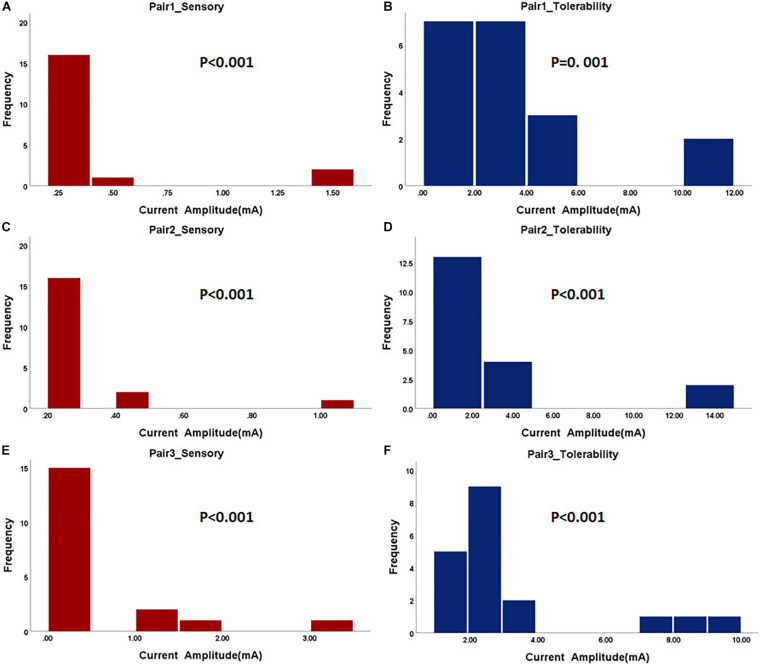
The histogram of sensory and tolerability thresholds of acupuncture electrode pairs. The *P*-value of the normality test is shown in each figure. P greater than 0.05 was considered as the normal distribution. **(A)** Sensory threshold of Pair 1. **(B)** Tolerability threshold of Pair 1. **(C)** Sensory threshold of Pair 2. **(D)** Tolerability threshold of Pair 2. **(E)** Sensory threshold of Pair 3. **(F)** Tolerability threshold of Pair 3.

The subjects rested for 30 min after the TENS stimulation to minimize the influence of sensations elicited in the first step. After that, the paralyzed side of the face was stimulated by EA. In a few cases, the distributions of sensory and tolerability thresholds of EA were skewed (*p* < 0.05) with substantial differences (Figure 5). The median value of the sensory thresholds was 0.2 mA in each pair of acupoints, and no significant differences were reported among the sensory thresholds of all pairs tested via Kruskal–Wallis H methods. The median values of the tolerability thresholds of Pair 1, Pair 2, and Pair 3 were 3.2, 2, and 2.4 mA, respectively. No significant differences existed among the tolerability thresholds of all pairs tested via the Kruskal–Wallis H methods. Table 4 shows the detailed statistical information of sensory and tolerability thresholds of EA. The tolerability thresholds were significantly greater than the sensory thresholds in each pair. The median, 25% quantile (Q1), 75% quantile (Q3), and outliers of the thresholds are shown in Figure 4B.

**TABLE 4:** The statistical sensory and tolerability threshold results of the electroacupuncture (EA’s) electrode pairs.

Threshold (mA)	Pair 1 (median [Q1, Q3])	Pair 2 (median [Q1, Q3])	Pair 3 (median [Q1, Q3])	*P*-value
Sensory	0.20 [0.20, 0.20]	0.20 [0.20, 0.20]	0.20 [0.20, 0.40]	0.329
Tolerability	3.20 [1.45, 3.90]	2.00 [1.20, 2.75]	2.40 [1.85, 2.95]	0.456
*p*-value	<0.001	<0.001	<0.001	

### The Correlation Between Current Intensity and Sensations

**FIGURE 6:**
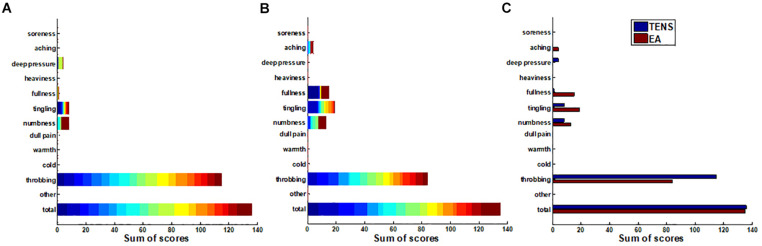
The sums of the modified Chinese version of Massachusetts General Hospital Acupuncture Sensation Scales (C-MMASS) scores of all the subjects. Each bar represented the sum scores at a descriptor of all subjects. In **(A,B)**, each color of a bar represented a score of one subject. **(A)** TENS sum scores. **(B)** EA sum scores. **(C)** Comparison of sum scores between TENS and EA.

Since the sensations perceived by the subjects varied from person to person, we applied a stack bar graph to depict the overall distribution of sensations measured by C-MMASS scores. In Figures 6A,B, each color of a bar represented a score of one subject. Figure 6A demonstrates the TENS-induced sensations including deep pressure, fullness, tingling, numbness, and throbbing; notably, throbbing was the most reported sensation during TENS stimulation. Figure 6B demonstrates the EA-induced sensations including aching, fullness, tingling, numbness, and throbbing; notably, throbbing was the most reported sensation during EA stimulation. In the comparison of C-MMASS scores of TENS and EA, aching, fullness, tingling, and numbness were more likely to be elicited by EA than TENS, and TENS-induced throbbing was higher than EA-induced throbbing (Figure 6C). The total scores were the sum of the scores of all descriptors, which quantified the total sensation of de-qi. Figure 6C shows that the total scores of TENS and EA were nearly similar.

**FIGURE 7:**
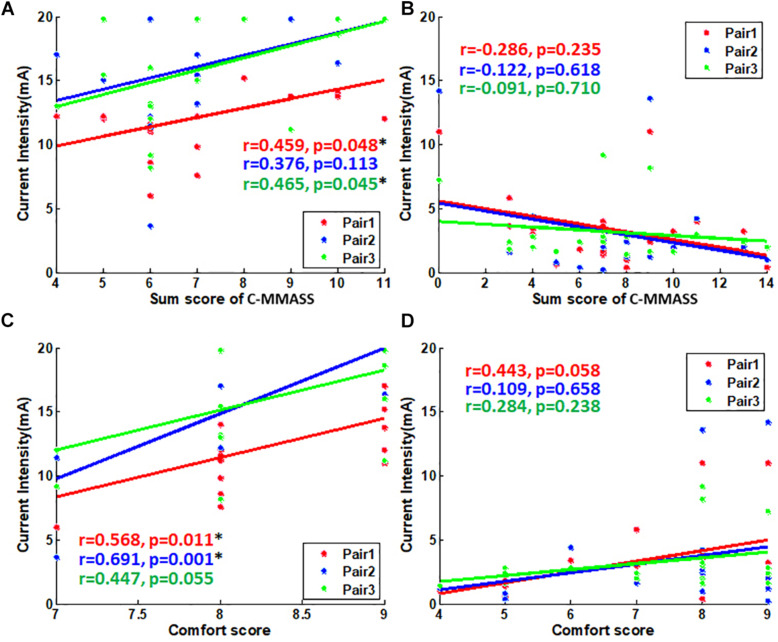
The scatter plot of scores vs. tolerability threshold. **(A)** The scatter plot of C-MMASS sum scores vs. TENS current intensities. **(B)** The scatter plot of C-MMASS sum scores vs. EA current intensities. **(C)** The scatter plot of comfort scores vs. TENS current intensities. **(D)** The scatter plot of comfort scores vs. EA current intensities. *P < 0.05.

All the scores were surveyed after the treatment at a tolerability threshold of 10 min. The relationship between tolerability thresholds and sensation scores was further evaluated. The total score of one subject was applied for correlation as it represented the overall de-qi sensation of the subject. The thresholds and total scores were correlated significantly in Pair 1 and Pair 3 (*p* < 0.05) during TENS stimulation (correlation coefficient, 0.46) (Figure 7A). However, thresholds and total scores were not correlated during EA stimulation (Figure 7B). In the analysis of correlationship between threshold and comfort score, the thresholds were significantly correlated with comfort scores in Pair 1 and Pair 2 (*p* < 0.05) during TENS stimulation (Figure 7C). However, the thresholds were not correlated with comfort scores during EA stimulation (Figure 7D). This suggested the association between both de-qi sensation and comfort with the current intensities in TENS stimulation.

### Finite Element Analysis

**FIGURE 8:**
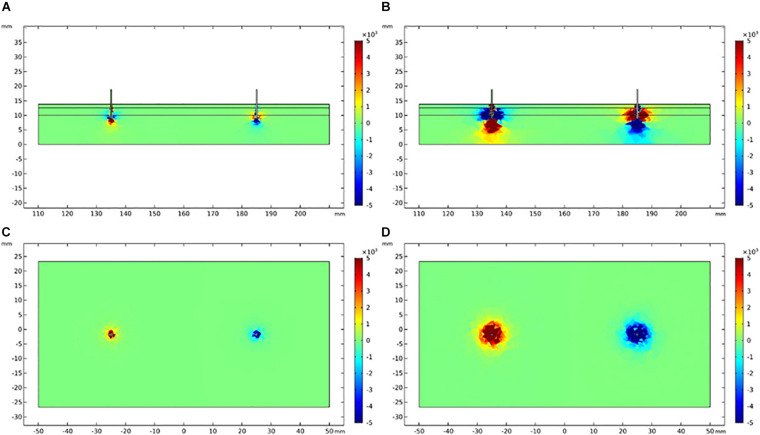
Electric field gradient distribution in TENS at different current intensity. **(A)** The longitudinal section of electric field gradient at 4 mA; **(B)** at 15 mA. **(C)** The cross-section (3 mm depth) of electric field gradient at 4 mA; **(D)** at 15 mA. Color bar indicates the magnitude of second order electrical field gradient (V/m^2^).

**FIGURE 9:**
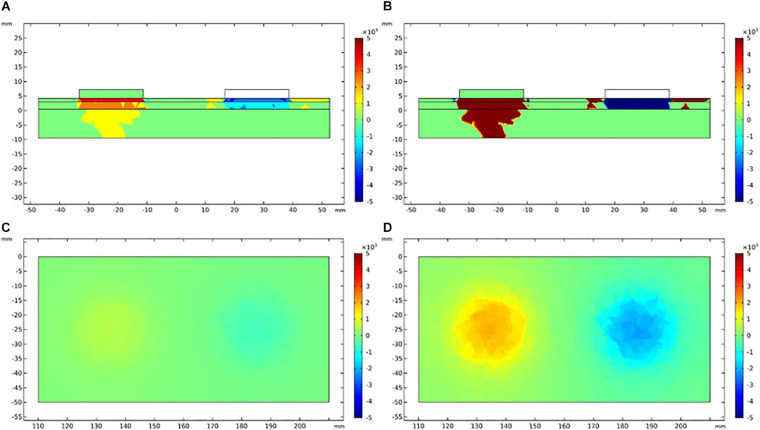
Electric field gradient distribution in EA at different current intensity. **(A)** The longitudinal section of electric field gradient at 0.2 mA; **(B)** at 2.5 mA. **(C)** The cross-section (3 mm depth) of electric field gradient at 0.2 mA; **(D)** at 2.5 mA. Color bar indicates the magnitude of second order electrical field gradient (V/m^2^).

The vertical and lateral distribution of electric field gradient was simulated and plotted to establish the possible activated areas of TENS and EA at specific current intensities, as depicted in Figures 8, 9. The color indicated the possibility of nerve activation, the bright color indicated a positive electric field gradient value, the dull color showed a negative electric field gradient. Bright regions showed membrane potential depolarization, and dull regions indicated membrane potential hyperpolarization. Electric field gradient distributions under the sensory and tolerability thresholds were plotted. The sensory threshold represented the minimum activated area, while the tolerability threshold showed the maximum activated area. In this study, we used 4 and 15 mA to represent the sensory and tolerability threshold of TENS, respectively. Besides, 0.2 and 2.5 mA denoted the sensory and tolerability threshold of EA, respectively.

The depth and diameter of the activated area were measured on the electric field gradient distribution map to quantitatively establish the activated area in TENS and EA. To define the activated area boundary, we applied a relatively small electric field gradient threshold with 100 V/m^2^ (Table 5). The activated depths of TENS and EA were similar at the upper threshold (tolerability threshold), whereas the activated depths were deeper in EA than TENS at the lower threshold (sensory threshold). The activated cross-section was larger in TENS than in EA. For instance, the activated cross-sectional diameter at 3-mm depth was over 43.3 mm in TENS and less than 20.3 mm in EA.

**TABLE 5:** The depth and a cross-sectional diameter of electric field gradient at different sensorial thresholds.

	TENS		EA	
	Depth (mm)	Diameter (mm)	Depth (mm)	Diameter (mm)
Sensory threshold	3.8	43.3	7	10.6
Tolerability threshold	13.8	50	13.8	20.3

## Discussion

In clinics, the current intensities during EA or TENS stimulation were gradually regulated to the level when skin shivering can be observed by the acupuncturist or at the patient’s maximal tolerable threshold, and the patient should not feel any discomfort (Zhou et al., 2011; Liu et al., 2017). In this study, the tolerability threshold was tested by gradually increasing the current intensity from 0 to 20 mA. Both TENS and EA were evaluated as comfortable since all the comfort rating scores were larger than 4. As such, we gave an exact current intensity during the treatment of Bell’s palsy patients by TENS and EA, which corresponded to the real clinical setting. In addition, the sensory threshold at which minimal sensations were perceived during stimulation was tested. By testing the sensory threshold, we established where the lower limit of the stimulation might be. The characteristics of these thresholds are listed as follows:

First, the thresholds varied significantly among different pairs of acupoints in TENS stimulation, but demonstrated no significant difference among different pairs of acupoints during EA stimulation. During TENS, the sensory and tolerability thresholds were significantly lower in Pair 1 compared with Pair 2 and Pair 3. Notably, electrodes of Pair 1 were located on the frontalis and partes temporalis, while electrodes of Pair 2 and Pair 3 were located on the orbicularis oris and masseter, respectively. The study of Ilves et al. (2019) found that the current intensity at which a forehead movement can be induced was significantly lower than the current intensity inducing the movement of the cheek and mouth, which concur with our findings. Such difference may be attributed to the thickness of the skin and subcutaneous tissues of different facial regions. Kim et al. (2019) measured the regional thickness of facial superficial adipose tissue. They demonstrated that the thickness tended to increase in the following order: forehead, temple, cheek, infraorbital, and perioral regions. Thicker facial adipose was related to a larger electrical conductivity of this tissue. However, this phenomenon cannot be explained by the thickness of the adipose layer fully. As the current controlled stimuli were applied, the compliance voltage can be increased to keep the current unaltered. More studies should be conducted on the distribution of synapses of sensory neurons of the face in the future to help in interpreting the sensory differences between the forehead and other parts of the face.

Second, the distribution forms of thresholds of different types of electrical stimulation were different. The thresholds of TENS were normally distributed, whereas the thresholds of EA were skewed distributed. The sensations elicited by TENS and EA were due to the activation of sensory nerve fibers in muscle (Wang et al., 1985; Beissner et al., 2010). The current spreading area of TENS was much larger than that of EA owing to the different shapes of the electrodes (Stux and Pomeranz, 1998). At the required activating thresholds, more sensory fibers may be activated by TENS. A larger sample size denoted a high probability of the normal distribution of the samples. Therefore, it can be inferred that the numbers of fibers activated by TENS were larger than those activated by EA.

Third, the sensory and tolerability thresholds of EA were substantially smaller than those of TENS. This may be attributed to: (i) The current density of TENS was smaller than EA. The pads used in TENS had a diameter of 22 mm, whereas the needles used in EA had a diameter of 0.3 mm. The current with pad spread to a larger area than the current with needles. The spread area of the electric field can be quantified by the results of our finite element analysis. At the sensory threshold of TENS and EA, the diameter of the electric field of TENS was almost four times the diameter of EA, whereas the depth of the electric field of TENS was almost 0.5 times the diameter of EA. Therefore, the area of the electric field of TENS was about eight times that of EA, and the sensory threshold of TENS was 20 times that of EA. At the tolerability threshold of TENS and EA, the diameter of the electric field of TENS was nearly 2.5 times the diameter of EA, whereas the depth of the electric field of TENS was similar to that of EA. Therefore, the area of electric field of TENS was about six times that of EA. The tolerability threshold of TENS was approximately six times that of EA. (ii) As the needle penetrates the skin, a deeper nerve stimulation was induced (Stux and Pomeranz, 1998). Therefore, the fibers stimulated by EA were different from the fibers stimulated by TENS. This may explain why the sensory threshold of TENS was much larger than that of EA, considering the size of their electric field. In contrast, the penetration depths of TENS and EA were similar at the current intensity of tolerability threshold; also, the tolerability threshold of TENS was similar to that of EA, considering the size of their electric field.

Although we quantitatively tested the current intensity according to the response of patients, the results should be varied if the pulse width and pulse repetition frequency were altered. The pulse width was 200 μs, and the pulse repetition frequency was 20 Hz in this study. In previous studies, Ilves et al. used a positive and negative phase duration of 400 μs and pulse repetition frequency of 250 Hz (Ilves et al., 2019), while Frigerio et al. (2015) used an average pulse width of 700 μs, and pulse repetition frequencies of 100–150 Hz. Of note, the total electrical charge delivered in the previous studies was nearly 25 times the charge delivered in our study under similar current intensity, stimulating time, and the size of the electrodes. Considering reports from the above two studies, the size of surface electrodes was similar to electrodes of TENS used in our study. At the current intensity of 2–4 mA, the range of sensory thresholds in the present study, Ilves et al. observed the facial movements at the forehead, cheek, and mouth regions (Ilves et al., 2019), while Frigerio et al. reported eye blink in acute facial palsy at about 4.6 mA in 55% of the participants (Frigerio et al., 2015). Herein, we found no facial movements during TENS at the tolerability threshold, while Ilves et al. found that the tolerability threshold was 7–8 mA (Ilves et al., 2019). Also, the mean tolerability threshold was about 12–16 mA in our study, which was 1.5–3 times greater than that in the study by Ilves et al. Notably, the results may be influenced by the types of the electrodes. Elsewhere, Zhou reported that (Zhou et al., 2015) the pain threshold of hydrogel electrodes and textile electrodes was different. Therefore, the sensory and tolerability threshold may also vary with different types or sizes of the electrodes.

For many years, the attempt to measure de-qi sensation quantitatively has developed a series of de-qi questionnaires (Kong et al., 2007; Kou et al., 2007; Yu et al., 2012). Some studies had also explored the de-qi sensations during EA or TENS stimulation (Zhou et al., 2011; Wang et al., 2013). However, reports on the relationship between de-qi sensations and the current intensities were rare. In this study, through correlation analysis, we explored whether the sensations were correlated with the stimulating current intensities among different subjects. From the results, the lower current intensities were related to lower total scores of de-qi sensations and higher scores of comforts during TENS treatment. However, this aspect was not significant during EA treatment. In the present study, the current intensities used for correlation analysis was the tolerability threshold. Therefore, two implications may be drawn from the results: First, the tolerable sensory was dependent on the current intensity linearly in TENS stimulation. As such, the de-qi sensations may be controlled quantitatively through regulation of the current intensity of TENS in the future. Second, the dose–effect relationship between the current intensity and de-qi sensations was not significant in EA. Compared with the result of TENS, it was suggested that the characteristic of stimulation by EA was specific and non-linear. The mechanism underlying this may be revealed by identifying which fibers or receptors were activated around the needle.

Finite element analysis refers to the assessment of a problem in engineering or mathematical modeling by establishing via a finite element model. The finite element model is established by dividing the problem domain into a collection of subdomains. Each subdomain is represented by a set of element equations to the original problem. Since it was first developed by Hrennikoff (1941) and Courant (1943), finite element analysis has received wide application in complicated engineering domains, including crash simulation, weather prediction, the simulation of electric fields in the human body, and so on (Blumhardt, 2001; Dutta et al., 2002; Filipovic et al., 2011; Datta et al., 2013). Currently, the finite element model can be established using commercial software such as COMSOL. When the complex problem is divided into small elements, the generated simulation results can accurately predict the distribution of the electric field, which is plotted in a fine map. Therefore, the current spread during the stimulation by TENS or EA can be easily simulated through finite element analysis. The spatial scope of TENS and EA stimulation at different current intensities can be viewed directly according to the simulation. Another potential application of the simulation includes calculation of the current density of electrical stimulation and establish the possible target fibers or receptors during the stimulation.

The quantitative investigation of stimulation current intensity was meaningful to understand the essential of de-qi sensations elicited by EA or TENS. The de-qi sensations, which are experienced as numbness, heaviness, soreness, and fullness are demonstrated to be associated with the activation of Aδ and C fiber (Wang et al., 1985; Beissner et al., 2010). The activation of sensory receptors of small afferent nerve fibers elicits de-qi sensations (Stux and Pomeranz, 1998). The peripheral nerve fibers are classified by size and conduction velocity (Kandel and Schwartz, 2013). The larger the nerve fiber diameter, the more rapid its conduction velocity speed. These different types of fibers are also connected with different sensory receptors (Kandel and Schwartz, 2013). For example, proprioceptors are innervated by large fibers such as Aα fibers, mechanoreceptors by moderate fibers such as Aβ and Aδ fibers, and nociceptors and thermoreceptors by Aδ and C fibers. According to the size principle, the electrical excitability of a nerve fiber is related to its size (Henneman, 1957). The action potential of a thicker nerve is lower than that of a thinner nerve. Therefore, during the stimulation by TENS or EA, the sensory receptors of thick fibers are inevitably activated before the de-qi sensations are experienced. It can be observed from our surveys on de-qi sensations that throbbing was the most frequently reported sensation during electrical stimulation (Figure 6). The sense of throbbing, which may indicate muscle spasm, was associated with the activation of thick motor fibers. As a contrast, the de-qi sensations elicited by manual acupuncture are mainly associated with the activation of small fibers (Aδ and C fibers) and their sensory receptors (Beissner et al., 2010). However, the sensations elicited by electrical stimulation can be altered by changing the type of electrode and stimulating parameters. The current density is influenced by the size of the electrode (Stux and Pomeranz, 1998). In this study, the current with a surface electrode spread a wider area than the current with a needle electrode. Therefore, the current intensity of de-qi sensations elicited by TENS was much larger than the current intensity of de-qi elicited by EA. Also, the magnitude of throbbing sense elicited by EA was relatively less than that elicited by TENS. We speculated that the reduction of throbbing sense that may be due to small fibers were more likely to be activated at the high-density electric filed. Therefore, although the clinical effectiveness of these two types of electrical stimulation was not compared, the de-qi sensations elicited by EA were closer to the manual acupuncture than the sensations elicited by TENS. Another critical parameter affecting de-qi sensations is the pulse frequency (Stux and Pomeranz, 1998). It has been demonstrated that stimulation with a low frequency (2–4 Hz) can release endorphins and cortisol (Groppetti et al., 2011; Brandon et al., 2014), which is critical to the effectivity of acupuncture anesthesia and anti-inflammatory (Stux and Pomeranz, 1998). High frequency (50–200 Hz) stimulation will make muscle spasm and has no time to relax between contractions, thus, preventing the achievement of de-qi sensations (Stux and Pomeranz, 1998). If the low-frequency stimulation is used, a strong stimulating intensity is needed to make sure the charge delivered to the fiber membrane is enough to activate the action potential (Stux and Pomeranz, 1998). However, the muscle contraction often becomes so vigorous if strong intensity is applied on the patients that they may not bear it, especially in the extremely sensitive regions of the body such as the face (Stux and Pomeranz, 1998). Therefore, a moderate stimulating frequency of 20 Hz was applied on the face in this study to balance the need of low frequency and low intensity. The studies on other parts of the body may use low frequency and high intensity to achieve more rigorous de-qi sensations in the future.

The achievement of de-qi is a prerequisite for the clinical effect of acupuncture according to the theory of traditional Chinese medicine (Yang et al., 2013). The studies on the mechanism of acupuncture analgesia had revealed how the activation of small afferent fibers (Aδ and C fiber) inhibits pain sensation (Stux and Pomeranz, 1998). The effect and mechanism of acupuncture on nerve regeneration is still unclear. Lu et al. indicated that a low-frequency (2 Hz) electrical stimulation was better than a high-frequency (10 Hz) electrical stimulation for nerve regeneration in the sciatic nerve of mice (Lu et al., 2008). Besides, the low current intensity (1 mA) was better than the high current intensity (4 mA) for nerve regeneration (Lu et al., 2008). According to these studies, mild stimulation by EA and TENS may be better than strong stimulation with de-qi sensations in the treatment of Bell’s palsy. More studies should be conducted on the effective dose of electrical stimulation on Bell’s palsy patients and the physiological characteristics under this stimulation dose. In our previous study, we had found that the recovery of Bell’s palsy was related to the symmetry of facial blood perfusion (Cui et al., 2016), and the acupuncture treatment with de-qi sensations had been demonstrated to be capable of regulating the blood flow (Pagani et al., 2006; Kim et al., 2016). Therefore, the studies on the effect of blood flow regulation of EA and TENS may be important in the investigation of their impact on nerve regeneration.

In summary, our study provided a new method to quantitatively explore the intensity and spatial scope of TENS and EA stimulation. This method will be helpful in the study of the mechanism on why a certain kind of electrical stimulation or a certain current intensity was effective in the clinic. Based on the sensation testing and finite element analysis methods, combined with the findings of anatomical tissue distribution around the acupoints, the activated neurons or target cells can be found out, and the key neurons or target cells that play an important role in the treatment can be revealed.

Being a pilot study, some limitations should be resolved in subsequent studies. First, we did not enroll healthy subjects, and the sensations may be altered on the paralyzed side of the face in Bell’s palsy patients, the sensory and tolerability thresholds measured may not represent the thresholds of healthy subjects. However, both TENS and EA are mainly applied on patients in clinics, which make our results meaningful in clinical practice. Second, the sample size was not inconclusive. We first reported the distribution of sensory and tolerability thresholds of Bell’s palsy patients stimulated by TENS and EA. The standard deviation of the measured thresholds may provide a basis for sample size calculation in the next study. Third, this was not a clinical study, and no conclusions have been made on the therapeutic effect of TENS and EA. Rigorous designed clinical trials are warranted to explore whether the current intensity at which de-qi sensations were elicited will be more effective than other stimulating intensities.

## Conclusion

This pilot study provided a new method for exploring the current intensity at which the de-qi sensations can be elicited by TENS or EA. The spatial distributions of electric field gradient at these current intensities were simulated via finite element analysis. The finite element analysis can, therefore, be applied as a simplified visualization tool to show the spatial scope of TENS and EA stimulation, such as stimulating depth and cross-sectional diameter. By modeling of tissues around acupoints comprising anatomical details of afferent nerve fibers, the finite element analysis will be helpful in investigating the stimulating dose at which de-qi sensations can be elicited by TENS and EA and its underlying mechanism. Additionally, the finding of the correlation between current intensity and de-qi intensity in TENS stimulation present a potential application of TENS on controlling de-qi sensations quantitatively. Nevertheless, the application of the method presented here should be conducted on more acupoints and a larger sample of subjects in the future.

## Data Availability Statement

The raw data supporting the conclusions of this article will be made available by the authors, without undue reservation.

## Ethics Statement

The studies involving human participants were reviewed and approved by the Ethical Committee of Shenzhen Traditional Chinese Medicine Hospital. The patients/participants provided their written informed consent to participate in this study.

## Author Contributions

HC, GL, and ZY conceived the presented idea. HC and HY performed the statistical analysis. WZ was in charge of evaluating Bell’s palsy subjects. XH supervised the findings of this work. HC, LW, YG, and XC carried out the experiments. LW, YL, YH, SZ, and MZ were in charge of recruiting Bell’s palsy subjects. HC took the lead in writing the manuscript. All authors discussed the results and contributed to the final manuscript.

## Conflict of Interest

The authors declare that the research was conducted in the absence of any commercial or financial relationships that could be construed as a potential conflict of interest.
